# Anthropological contributions to historical ecology: 50 questions, infinite prospects

**DOI:** 10.1371/journal.pone.0171883

**Published:** 2017-02-24

**Authors:** Chelsey Geralda Armstrong, Anna C. Shoemaker, Iain McKechnie, Anneli Ekblom, Péter Szabó, Paul J. Lane, Alex C. McAlvay, Oliver J. Boles, Sarah Walshaw, Nik Petek, Kevin S. Gibbons, Erendira Quintana Morales, Eugene N. Anderson, Aleksandra Ibragimow, Grzegorz Podruczny, Jana C. Vamosi, Tony Marks-Block, Joyce K. LeCompte, Sākihitowin Awâsis, Carly Nabess, Paul Sinclair, Carole L. Crumley

**Affiliations:** 1 Department of Archaeology, Simon Fraser University, Vancouver, British Columbia, Canada; 2 Department of Archaeology and Ancient History, Uppsala University, Uppsala, Sweden; 3 Department of Anthropology, University of Victoria, Victoria, British Columbia, Canada; 4 Hakai Institute, Heriot Bay, Quadra Island, British Columbia, Canada; 5 Department of Vegetation Ecology, Institute of Botany of the Czech Academy of Sciences, Brno, Czech Republic; 6 School of Geography, Archaeology and Environmental Studies, University of the Witwatersrand, Johannesburg, South Africa; 7 Department of Botany, University of Wisconsin–Madison, Madison, Wisconsin, United States of America; 8 Institute of Archaeology, University College London, London, United Kingdom; 9 Department of History, Simon Fraser University, Vancouver, British Columbia, Canada; 10 Department of Anthropology, University of Maryland, College Park, Maryland, United States of America; 11 Department of Anthropology, Rice University, Houston, Texas, United States of America; 12 Department of Anthropology, University California Santa Barbara, Santa Barbara, California, United States of America; 13 Polish-German Research Institute, Adams Mickiewicz University in Poznań, European University, Viadrina, Poland/Germany; 14 Department of Biological Sciences, University of Calgary, Alberta, Canada; 15 Department of Anthropology, Stanford University, Stanford, California, United States of America; 16 Independent Scholar, Seattle, Washington, United States of America; 17 Department of Geography, Western University, London, Ontario, Canada; 18 Atlohsa Native Family Healing Services, Canada, London, Ontario, Canada; 19 Department of Anthropology, University of Northern British Columbia, Prince George, British Columbia, Canada; 20 Department of Anthropology, University of North Carolina-Chapel Hill, Chapel Hill, North Carolina, United States of America; 21 Integrated History of Future of People on Earth (IHOPE) Initiative, Uppsala, Sweden; National and Kapodistrian University of Athens, GREECE

## Abstract

This paper presents the results of a consensus-driven process identifying 50 priority research questions for historical ecology obtained through crowdsourcing, literature reviews, and in-person workshopping. A deliberative approach was designed to maximize discussion and debate with defined outcomes. Two in-person workshops (in Sweden and Canada) over the course of two years and online discussions were peer facilitated to define specific key questions for historical ecology from anthropological and archaeological perspectives. The aim of this research is to showcase the variety of questions that reflect the broad scope for historical-ecological research trajectories across scientific disciplines. Historical ecology encompasses research concerned with decadal, centennial, and millennial human-environmental interactions, and the consequences that those relationships have in the formation of contemporary landscapes. Six interrelated themes arose from our consensus-building workshop model: (1) climate and environmental change and variability; (2) multi-scalar, multi-disciplinary; (3) biodiversity and community ecology; (4) resource and environmental management and governance; (5) methods and applications; and (6) communication and policy. The 50 questions represented by these themes highlight meaningful trends in historical ecology that distill the field down to three explicit findings. First, historical ecology is fundamentally an applied research program. Second, this program seeks to understand long-term human-environment interactions with a focus on avoiding, mitigating, and reversing adverse ecological effects. Third, historical ecology is part of convergent trends toward transdisciplinary research science, which erodes scientific boundaries between the cultural and natural.

## Introduction

Historical ecology is a field of inquiry that has come of age and currently finds itself at a crossroads. After decades of interrelated developments in both ecology and archaeology, historical ecology is increasingly recognized as an inclusive intellectual hub for exploring a range of fundamental questions in disciplines such as ecology, biology, archaeology, anthropology, history, geography, and ethnobiology. The term is increasingly cited in academic literature and researchers are beginning to use the label to identify themselves [1–6]. The appeal of historical-ecological research is that it operates on multiple temporal scales and across disciplinary boundaries that have long separated the social and natural sciences [1,7]. It also generates applied research questions and data for historically grounded and socially just conservation programs, in which environmental initiatives consider the totality of human-environment interactions and foster a critical awareness of the imposition of “green” policy on communities, many of whom may be marginalized [2,8–10].

Historical ecology, however, is not organized around a single unified methodology or theory, and there are no dedicated publication venues. Indeed, most publications in historical ecology showcase the crossover potential of ecologically and socially engaged historical research. There are many points of departure that lead researchers to engage with historical ecology. At the core of many historical-ecological research initiatives is a recognition of the interpretive potential of combining archaeological, historical, and ecological data and expertise [11,12]. Based on our research focus, two “types” of historical ecology appear to have formed, primarily associated with either archaeology or ecology, and resulting in a parallel but largely non-overlapping literature [6,13–17].

Szabó [6] contends that historical ecology is in a third scholarly iteration, which will either stay nestled under a multidisciplinary umbrella or emerge as a conventional (institutionalized) academic discipline. Whether or not the concept is bound to either trajectory, this moment is cause for reflection on past, present, and future research questions of its practitioners. Rather than struggle with the circuitous task of defining historical ecology, we instead employed crowdsourcing and consensus-building methods to identify research questions guiding current historical-ecological research, as well as questions which may become more important in years to come.

This research initiative grew out of a symposium held at Uppsala University in 2014. Researchers identifying as historical ecologists presented their work, and while seeking to frame a discussion about the future of historical ecology, realized that there were gaps in our cumulative perceptions on the current state of the research program. In order to better understand who historical ecologists are and what they do, we crowd-sourced over 300 questions in a priority-setting initiative between November 2014- November 2015. Questions were edited down to 50, which we determined was a suitable and unconstrained number and comprised a representative subset of the total pool of submissions.

## Methods

For identifying and ranking important questions in historical ecology, an open, inclusive, and consensus-driven four-stage group process was followed. This process builds on similar methods used by Sutherland and colleagues’ numerous priority-setting research works [18–21] as well as the research of Seddon et al. [22], Kintigh et al. [23], and Parsons et al. [24] in paleoecology, archaeology, and marine biodiversity, respectively. Social justice organizing tactics, in which challenging conversations are facilitated to catalyze breakthrough thinking and lasting agreements, also inspired the process.

In 2014, an international meeting was convened in Uppsala (Sweden), focusing on early-career researchers working in historical ecology. Twenty-eight participants came together to generate discussion on key topics and share perspectives on the various “types” of historical ecology. A global survey was then circulated online to further crowdsource research questions from academics working broadly under the historical ecology umbrella. Over 300 questions were submitted and sorted into two or more of the following nodes: (1) climate and environmental change and variability; (2) multi-scalar, multi-disciplinary; (3) biodiversity and community ecology; (4) resource and environmental management and governance; (5) methods and applications; and (6) communication and policy.

**Fig 1:**
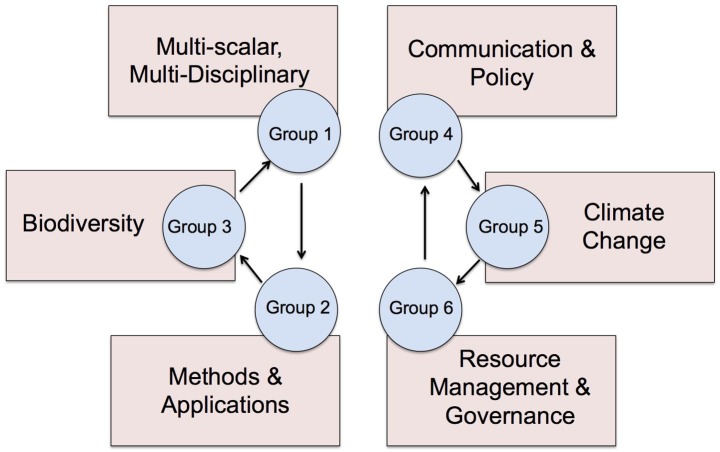
Consensus-Building in Second Workshop. After sorting questions into each group (10–12 participants and a facilitator) are allotted 90 minutes to select the questions most pertinent to the node. The group then rotates to the next node.

**Fig 2:**
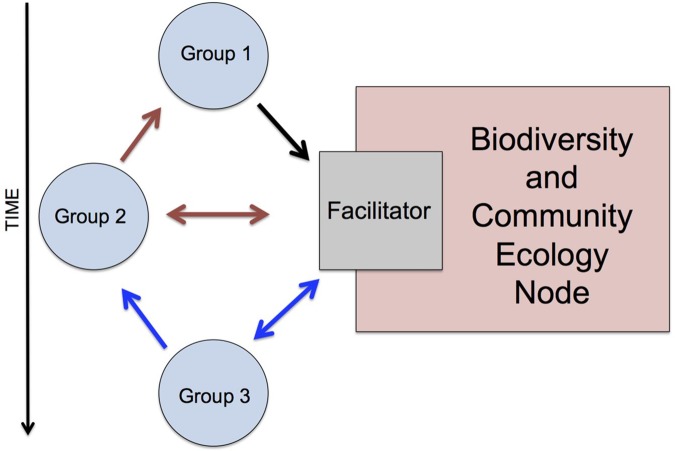
Facilitation Process Example. The facilitator stays with the same node throughout the day and discloses results and insights from previous groups’ discussion with each new group. For example, Group 1 works through the list of biodiversity and community ecology questions with the help of a facilitator. Group 2 then works with the same question list but is able to interact with Group 1’s ideas (but not vice versa) through the facilitator. Group 3 works with the original list again but is exposed to compounded ideas from Groups 1 and 2 through the facilitator, and so on until each group has an opportunity to discuss all six nodes.

In 2015, a second-stage meeting convened in Vancouver (Canada). Initially, 47 workshop participants from various disciplinary backgrounds, all self-associated with historical ecology, broke into groups to discuss and select preferred questions from the six nodes (Fig 1). Priority questions were identified from every theme in a consensus-based process, inspired by social justice organization in which discussion is supported by impartial peer facilitators. Rather than simply voting for questions, we used a consensus process that emphasized maximizing group intelligence through discussion and debate. The consensus method aims to eradicate a “tyranny of the majority” that occurs in democratic voting and allows for more opinions to be considered. Facilitators were chosen based on their experience in organized facilitation roles; for example, three group members had previously attended extensive facilitation-training workshops in social justice settings. These facilitators trained the other three volunteers, and after every node, would regroup to exchange techniques and group-organizing ideas. Facilitators refrained from influencing discussions, and concentrated on ensuring groups stayed on time and on topic, to maximize creative cooperation and to ensure group memory (Fig 2). A total of 82 questions resulted from this deliberative process.

In a third stage, a subset of workshop participants (53%) volunteered to edit the entire list of 82 questions that had been identified as the most important and relevant by deleting duplicates, fusing similar entries, and ensuring that the wording of the questions was adjusted to reflect the views of participants in the second stage. With 62 questions remaining, workshop participants decided to open a final editing round to our online community of scholars. In the last stage, the online community, comprising all participants from both the Uppsala and Vancouver workshops, were invited to rank the remaining short list of questions on a scale of importance (1–5) to reduce the number of questions to 50. We chose the number 50 because we found it was a large enough number to cover the depth and breadth of historical ecology, but small enough to force participants to think about the most pressing issues. In this online ranking forum, participants were invited to give feedback on the process and provide further comments on the formatting and content of questions.

A final quality-control round was a consensus-driven process amongst workshop organizers (from the New International Community of Historical Ecology–NICHE). This editing session was necessary for unanticipated outcomes that occurred throughout the online ranking exercise. For example, four questions were tied in the ranking for “last place”. Using the survey output and the facilitators’ minutes from the workshops (four facilitators were present in this final stage), we collapsed sufficiently similar questions, and re-organized their place in the various nodes. The wording of three questions was subsequently adjusted slightly in light of reviewers’ comments on a first draft of this paper. Ethics approval was granted specifically for this study by Uppsala University Ethics Board on behalf of the State Research Council.

### Limitations

The range of questions developed through this process reveal limitations in both the audience responses and the scope and scale of topics selected. During the first stage of the process, many participants preferred broad questions, seeing them as more inclusive, relatable, and relevant for defining “grand challenges” in a multi-disciplinary endeavor c.f. [25]. However, those seeking testable hypotheses and more explicit research trajectories often preferred specific questions. The final ranking survey reflects the overwhelming participant preference for broad questions—specific questions were favored only when they were vigorously defended in-person during the workshops.

As can often be expected when attempting to generate collective knowledge, the online ranking of questions was methodologically imperfect and preliminary divisions of questions into themes are likely to have limited lateral and creative thinking, as demonstrated in other similar projects [20]. Feedback submitted by participants in the online ranking exercise revealed frustration over the perceived similarity of some questions, vagueness of wording, and disproportionate representation of certain research topics. We therefore decided to discuss rankings and debate them in person rather than stick to the exact output (e.g., marine-based questions were ranked lower because demographically, more researchers study terrestrial ecosystems, but that does not make marine questions less important). The final version of the quantitatively ranked question list incorporates some adjustments in response to the qualitative concerns of online participants, and reflects the discourse and debates generated during the workshops.

Although the initial list of questions submitted online represented engagement with a wider variety of disciplinary and geographical backgrounds, the meetings were disproportionately attended by individuals affiliated with ecological/environmental anthropology and archaeology, and to a lesser degree with participants from ecology, biology, and geography. While calls for participation went out widely and were formulated with inclusivity in mind, the process favored English-language speakers and there was obvious and regrettable under-representation from strands of historical ecology in South America (e.g., Arqueologia e Ecologia Histórica dos Neotrópicos), Germany [26], France [27,28], Russia [29], and Japan [30]. Biases in representation reflect the limitations of the communities engaged and the challenges of networking across linguistic, geographic, and disciplinary divisions.

## Results

The initial open-call for research questions led to over 300 submissions from 20 countries. The majority of respondents (71%) were affiliated with Canadian, Swedish, American, and UK institutions, with lesser representation from individuals in Brazil, Colombia, Poland, Belgium, China, Mexico, the Netherlands, France, Zimbabwe, and South Africa. The majority of respondents pursued or held PhDs (89%) and were employed by, or pursuing degrees at, academic institutions.

The goal of the exercise was to set priority or key research questions among participants, recognizing that (1) developments in historical ecology and its meanings differ across disciplines and geographical context, and (2) questions were inspired by researchers more heavily engaged on the anthropological and archaeological spectrum of historical ecology. As such, our results reflect the strong anthropological background and limited geographic scope of our participants. This does not lessen the strength of the exercise or importance of the research. Firstly, the anthropological slant will serve to address those in the natural sciences who may acknowledge that humans cannot be removed from their long-lived landscapes, but struggle with how to reconcile this influence in their research [11,31]. Secondly, those working in countries that have a rich history of coupling the natural and social sciences (Egypt, India, Greece), but were not represented, can borrow, contrast, model, critique, be inspired by the diverse list of questions that can be reframed in many global contexts.

In the following sections, questions are divided by thematic node, each with a segment clarifying or detailing the relevance of the question set. There is no implied rank given to the numbered questions, but they are discussed in a logical order of applicability to each chosen topic. We conclude by highlighting three major themes that emerged from the process of this priority-setting exercise.

### Climate and environmental change and variability

Many have advocated for a new geological epoch, the Anthropocene, on the basis that human forces, for the first time in history, now operate at a global scale comparable in effects to geophysical and climatic processes [32–35]. It has been argued that the scale and agency behind climate change today is unique within the longue durée of human history. However, there is a need to distinguish between large-scale climate-forcing mechanisms and inherent small-scale climate variability affecting human lives, the latter of which can be both produced and mitigated by people [36,37]. Both of these concerns are reflected in the first set of questions.

1. What roles have humans played in extinction events and what can we learn about these large and small-scale changes?

2. When did human activities begin to have significant impacts/effects on their environments?

3. What factors allow human populations to become more decoupled from immediate environmental constraints?

4. What are the archaeological proxies of past climatic stability or instability?

5. What are the effects of climate and environmental change on human health and disease?

6. How predictable are human responses to environmental change, and how can we model such responses for future planning?

7. How is climate change affecting the management of eco-cultural and geo-cultural heritage?

8. How did past societies respond to sudden environmental shocks (e.g., extreme weather) and what can we learn from this?

9. What factors have made some communities more adaptable to environmental change than others?

At least two of these questions (8, 9) are explicitly concerned with past human experience of environmental variability and highlight the need to develop established research directions exemplified in ethnoecology [38–40], while a third question (5) lends itself implicitly to a historical approach, as illustrated by studies looking at the past interactions of climate, disease, and society [41]. Question 3 addresses the modern phenomena that allow human societies to operate, at least temporarily, beyond environmental constraints, for better or worse.

Question 2 raises the controversial issue of reclassification of the current geological epoch as the Anthropocene. The timing of the onset of this proposed epoch is widely debated [32,34,42,43]. Although some workshop participants were reluctant to use the term Anthropocene, inclusion of related questions illustrates the desire of many researchers in historical ecology to continue drawing attention to longer-term processes of human engagements with the environment and our “entry into the planetary machinery” [15]. The formulation of Questions 3–5 was widely debated in the group. On one hand, these formulations should be seen as transitory, reflecting the problems of representing the mutual dependency of humans and environment. On the other hand, they are indicative of the challenge of interpreting and dealing with the multi-scalar interdynamics of stability and change in the archaeological, geomorphological, and paleoecological records.

Questions 1 and 6–9 emphasize how historical-ecological data can be used to address contemporary issues of climate and environmental change. It has been highlighted that historical data offer a vital contribution as we plan for and mitigate future climatic change [38,39,44,45]. Question 7 takes an alternative perspective, asking how such changes might affect our ability to preserve eco-cultural and geo-cultural heritage. This drive toward applied research [46,47] is at the heart of historical ecology’s engagement with issues of climate change.

### Multi-scalar, multidisciplinary

A key strength in ecology, ethnoecology, and archaeology is an interest in multi-scalar perspectives that incorporate a wide range of data sources. Workshop participants were keen to continue integrating multi-scalar perspectives into historical-ecological research, captured in Questions 10–14, which relate to research methodologies and frames for engaging with multiple scales and the complex indices that represent them. Question 15 addresses more practical and ethical issues related to the research processes of historical ecology.

10. How do historical ecologists address different temporal and spatial scales, how do we define/communicate them, and how do we study their interactions?

11. How can archaeological and ecological methods be standardized for evidentiary and temporal comparability?

12. Does historical ecology relate exclusively to the longue durée, or “deep time” and, if so, how should those concepts be defined?

13. How do we constitute humans as integral parts of ecosystems and how do we conceptualize humans as one of many species in an ecosystem? At the same time, how can environmental history, in which humans are always regarded to be the protagonists of ecosystem change, effectively cooperate with historical ecology, which regards humans as one of the many species in an ecosystem?

14. How do we engage with the concept of sustainability in historical ecology, especially given constantly changing environmental dynamics, with or without humans?

15. What data standards should we develop to aggregate relevant information in a consolidated open-source database?

Question 10 highlights the familiar challenge of working with multi-scalar perspectives. Historical ecologists make particularly effective use of the extended spatial and temporal scales represented in paleoecological and archaeological datasets [48–50]. However, practitioners also grapple with the difficulties of combining fragmented datasets of varying degrees of temporal resolution [51,52], and resolving localized versus regional landscape dynamics. In geography, temporal and spatial scales have long been discussed, particularly since the “spatial turn” introduced spatiality as a frame of analysis [53–55]. Historical ecology can deal more competently with the spatial and temporal continuities and variability that characterize complex landscape processes by researching the connections and interdependencies of variables (similar to the concepts of pattern and process in ecology discussed below).

Question 11 addresses a concern about the perceived incompatibility of methods and temporal and spatial scales used in archaeology, anthropology, and ecology/paleoecology, as noted almost a decade ago by Bailey [52]. However, this question may reflect less of a methodological challenge than a challenge of integrating conceptual frameworks and goals of various specialized fields [56]. Question 13 reflects a concern for cognitive dissonance between researchers whose theoretical and epistemological frameworks may be said to align with either the social or the natural sciences. Global environmental change research was pioneered by natural scientists, yet humanities research situates people as the medium through which environmental change not only occurs, but is also experienced. Environmental humanities furthers a critical engagement with the construct of nature and examination of global power relations in which sustainable development operates [57,58], amongst other contributions.

Multidisciplinary projects are sustained with good collaborative relationships [59], and communication can often be a determinant of successful multi-disciplinary work [60]. Language may be the point of divergence for those from different research traditions; for instance, we found that “longue durée”, “deep time” (Question 12), and “sustainability” (Question 14) have a multitude of associated meanings, suggesting a need for consensus building when defining our terms.

Question 15 draws attention to developing standards for merging different types of data in open and accessible ways. The tendency to prioritize data generation over data curation and publication must be addressed [61]. The INTIMATE group is one example of a successful database initiative, facilitating the Integration of Ice core, Marine, and Terrestrial paleoclimate proxy records for North Atlantic and Australasian regions [62]. However, the INTIMATE group example is noticeably lacking in collaboration with social scientists, again reflecting an exclusively quantitative focus that some workshop participants found worrisome. While model-based projections of climatic change and variability powerfully inform future environmental planning, they can inadequately incorporate smaller-scale anthropogenic activity or human political responses and governance [63–65].

### Biodiversity and community ecology

Historical ecology is uniquely poised to assess the decadal, centennial, and even longer-term landscape consequences of human activities, as well as more subtle anthropogenic changes to ecological communities and populations e.g. [66–73]. Insights from this research may prove valuable in future decision-making regarding the management of resources and the management of people vis-à-vis resources. For example, Anderson [39,74] provides case studies of how resource management is constructed within religious and ethical codes. Such research can also be valuable for critiquing dominant narratives that continue to shape policy and management interventions based on assumptions about the causes of environmental degradation [75]. A strength of the historical-ecological research program is its ability to contextualize biodiversity as both an ecological and social concept. Biodiversity is a scientific field of inquiry, as well as a social construct, political referent, and topic of moral discourse (see [76]).

Historical-ecological research examines the interconnections between climatic and biotic variability (such as wildlife grazing, browsing, and fire) in the context of human land use and management [77–84]. For example, human-mediated biological invasions often initially increase local biodiversity while decreasing differences between sites [85]. Over time, inclusion of invasive species in a location may lead to an ultimate decrease in local diversity [86,87]. Questions 16–21 all relate to the dynamics between plant and animal species and humans in a given landscape.

16. How does the removal or introduction of species affect landscape and seascape ecology?

17. What is the potential of evolutionary history and paleoethnobotanical knowledge for plant conservation?

18. What is the relationship between past human activities and changes in the morphology and/or phenotypic traits of plant and animal species?

19. To what degree can we use ancient and modern genetic data (e.g., genetic structure) to infer past and present management practices and human influences on other species and populations?

20. How did anthropogenic land-use practices structure vegetation patterns prior to c. AD 1500 and the commencement of European global expansion?

21. When are “invasive”, “native”, and “introduced” useful concepts? Should these terms be applied to humans as well as to other species?

Humans are conscious architects of biodiverse communities [40,73,88], but such actions can also be interpreted as producing “epiphenomenal effects” on community ecology [89–91]. Practices like terracing, diking, transplanting, pruning, coppicing, livestock penning, broadcast burning, and fertilizing shape landscapes and associated community compositions in intentional and unintentional ways [40].

In addition to the more widely cited impacts of agricultural and pastoral pursuits on landscapes, human activities have also directly shaped ecosystems in intertidal and aquatic environments. For instance, some rock alignments that were once thought to be naturally occurring in intertidal marine environments now prove to be ancient remnants of large marine enhancement projects that expanded habitat and productivity and enhanced in marine resource availability [92]. Other human reactions to marine resource instability warrant further investigation (see Question 26) including substitution of aquatic foods for terrestrial alternatives, reduction of harvests and augmentation of resource pools [93,94], and mitigating, and seeking food sources more broadly [95].

Smaller scale anthropogenic changes to ecological communities can be detected at the molecular level, as addressed by Questions 18–19, which acknowledge the use of phenotypic and genotypic markers for investigating past human-environment interactions. Researchers commonly use contemporary plant community assemblages to infer the presence of past human ecosystem modifications [73,96–98], but genetic and/or morphological differences are increasingly used indicators [70,99–101]. Mutualism, co-evolution, and hybridity are key concepts in analyzing interactions between species and landscapes that can be better utilized to predict how communities might change with anthropogenic modification. Human niche construction models offer further modes of inquiry in the study of eco-cultural dynamics [16,102,103], and relate to important concepts such as landscape domestication [104], novel ecosystems [16] and neobiotic species [34].

Because mutualism, co-evolution, and human niche construction take place on varying timescales [105–107], it is relevant to consider the temporal baselines used to understand the interplay between agents and processes in landscape formation. Question 20 refers to the use of appropriate baselines to better appreciate rates of change and scales of impact brought on by human transformations of landscapes. Such baselines are treated as transitory, rather than static, but allow us to frame important research questions that can be contingent on historical circumstances. Related to this discussion is Question 21, which asks when and if the idea of “invasive species” is a useful concept. Some would argue that the terms “invasive” and “alien” are normative and culturally freighted [108], imposing ideals of authenticity and stasis, or that invasive species eventually stabilize as a new normal [109]. Most workshop participants reasoned that because invasive species cause realized and measurable disturbances (changes) to otherwise diverse socio-ecological systems, such terms should remain an important frame of reference and field of inquiry [110,111].

Worldwide, many of the most biodiverse landscapes have been produced by long-term, anthropogenic management practices [112]. In places where local management has been discontinued, or cultural landscapes have become relict, historical-ecological research has demonstrated that the absence of humans can lead to a decline in species diversity, landscape heterogeneity, and threats to livelihood [45,113–117].

22. What are the ecological impacts of anthropogenic soil (and sediment) transformations?

23. How has anthropogenic broadcast fire influenced ecosystem dynamics?

24. How do we identify different cropping styles (e.g., monocropping, polycropping, perennial orcharding) in the past and their effects on ecosystems and landscapes?

Questions 22–24 are related to the anthropogenic modification of soils and vegetation through fire and/or building of soils. Much attention has been given to the formation of *terra preta* or “Dark Earths”, created through the combination of infield and fallow burning, composting, and mulching. Anthropogenic soils have been shown to harbor important reservoirs of agro-biodiversity [118–122] and enhance forest productivity [123–125]. However, in many landscapes the customary practices that shape such soils are now discontinued. Historical ecology offers tools to understand not just the ecological dynamics of soil modifications and fire use, but also the social organization and cultural significance of these practices and the landscapes they produce [126].

Broadcast fire is an essential tool in landscape management, both to create mosaics of successional stages and to reduce wildfire spread. Seasonal fires and burning of different vegetation types creates and encourages vegetation mosaics and also protects forest patches (see for instance [120,127–129]). Recent studies in western North America have combined paleoecological and archaeological data to examine changes in anthropogenic broadcast fire regimes and found notable changes with the onset of Spanish colonialism [130,131]. Historical anthropogenic fire regime data can also inform contemporary fire and land management policies, such as in the West Arnhem Land Fire Abatement (WALFA) project, which now incorporates and restores Indigenous fire management practices [132,133].

Barthel et al. [134] (see also [135]) referred to continuing cultural landscapes as bio-cultural refugia where local knowledge, species, techniques, and methods are crucial for both biological and agro-ecological diversity (see Question 24). Other customary settlement practices, including those relating to waste discard and manuring, may also have beneficial ecological consequences that go well beyond the epiphenomenal, and could be conceived as central to situational processes of landscape domestication [136,137].

While the direct effects of resource extraction, industrial pollution, and land-use change on biodiversity are often well characterized, subtle and indirect consequences of low-impact cultural practices have yet to be investigated, prompting the following questions.

25. How and why does biological diversity (e.g., alpha and/or beta diversity) correlate with proxy indicators for cultural diversity (e.g., linguistic diversity)?

26. How have people responded to temporal-spatial fluctuations in marine resources?

27. What are the environmental impacts of political and economic restrictions to—or increases in—human mobility?

28. What are the environmental effects of past conflict and military activity?

At the core of historical-ecological research is the understanding that culture, in terms of local knowledge, customary practices, traditions, social norms, and languages, is intimately linked with landscape. Question 25 highlights an interest to further document and understand this integration [138]. Question 26 draws attention to the fact that human influences on marine environments also need consideration [12,139], prompted in part by concerns about the impacts of future sea-level rise [140] on the livelihoods of coastal people [141], the changes in habitat distribution and ecosystem functions that may ensue [142], and also the likely impacts on coastal heritage [143].

Changes to human mobility on different spatial scales (see Question 27) may alter population densities and thereby concentrate resource use [144,145]. For example, sedentism and land loss for pastoral peoples has implications for grazing intensity and grassland composition [146,147]. Constrained mobility may impact cultural practices tied to landscapes, though in other cases international and jurisdictional barriers may isolate kin networks and communities in ways that result in the discontinuation of local traditions [148], including resource management practices [149]. Conversely, limited human mobility may reduce access to resources, increase exposure to pollutants, and produce other negative human impacts [150,151].

The environmental impact of warfare, political upheaval, and human conflict is also becoming a key line of historical-ecological research (Question 28). The ecological impacts of modern weaponry—including the testing and use of nuclear weapons—can be demonstrated [152–154]. However, it should be pointed out that an under- acknowledged aspect of historical warfare in the early modern period is their influence on contemporary landscapes both in environmental impact [155] and heritage status commemoration.

### Resource and environmental management and governance

Practitioners of historical ecology share an interest in resource management and governance. This concern arises from an interest in applying knowledge to the politics of conservation and sustainability and to bring this to the forefront in discussions of environmental history and resource conservation. Governance and applied policy is at the core of many of the research questions, with the growing recognition that the best paths toward resource management depend less on managing resources and more so on managing people [39,156]. These concerns relate to a heightened understanding of the geopolitics and differential equities in which conservation and sustainable development initiatives operate[157].

This cluster of questions emphasizes dissolution of human-nature binaries and represents an embrace of multiple ways of knowing. Historical ecologists are aware of the ties between landscape ecologies and issues of human economy, well-being, sociality, and spirituality. The research insight provided by historical ecology to resource managers is therefore not simply an exercise in expert data exchange, but a meaningful, emergent process that aims to bring about collaboration and co-management strategies between people from different backgrounds [158]. Questions 29–38 reflect the importance of a plurality of perspectives for addressing social and environmental challenges.

29. How are past relationships between centers and peripheries (e.g., urban centers and hinterlands) characterized in terms of resource management and governance?

30. Why do different cultural groups in the same bioregions utilize resources in dissimilar ways?

31. What are the correlations between the health and well-being of humans and perceived status of the ecosystems they rely upon?

32. How do traditional resource management practices of migrant human populations shape newly encountered land- and seascapes?

33. What is the role of geopolitical power in the development, maintenance, and dissolution of cultural ecosystems?

34. How has the construction of borders, boundaries, and frontiers affected land-use practices?

35. How have people altered and managed their land- and seascapes to enhance desirable resources in coastal regions?

36. What has been, and continues to be, the impact of imperialism and colonialism on cultural ecosystems?

37. How can we best engage with Indigenous and/or local communities to respectfully incorporate traditional and local knowledge into historical ecology projects that are specific to place?

38. How can historical ecology address current and future challenges of global food sovereignty and security, both in terms of geopolitical constraints and sustainable ecological practices?

This research node identified issues of human health and well-being (Question 31) and food sovereignty (Question 38) as themes that require an applied focus. Workshop participants also signaled concerns with the contested and tentative nature of human relationships with landscapes, ecosystems, or resource bases. Many wanted to see critical research into the construction and (sometimes coercive) maintenance of geopolitical borders (Question 34) and centers and peripheries (Question 29) and/or “frontiers” [159,160]. The challenges of transboundary environmental management and protection have been gaining recognition in recent decades [161]. It is clear that cartography and socio-economic negotiations of power have relevance to local, traditional, and Indigenous governance systems and deserve research attention [162,163].

The legacies of imperialism and colonialism (Question 36) and global power dynamics more generally (Question 33) have ongoing ramifications for how resources are managed and governed and provide key contextual dimensions to our understanding of the recent past and present [164]. Many historical ecologists from social science backgrounds concentrate on how oppressive states, top-down management systems, or hyper-extractionist capitalism have been and are increasingly affecting global resource bases. We argue that an applied historical ecology is obligated to acknowledge and scrutinize such concerns and contribute to the mitigation of unjust practices [165]. Other social scientists have developed diverse tactics and tools for evaluating the efficacy and sustainability of resource management and governance [166].

Globally, the increasing commodification of biophysical resources and human labor has caused the disenfranchisement of local or Indigenous people and communities [159,167–169]. Diminishing Indigenous and local control of, and engagement with, lands and waters, depended on for material and spiritual sustenance, has led to concomitant reductions in cultural and biological diversity [39,170] as well as exploitative mismanagement of resources [171]. Questions 30, 32, and 37 deal with the use of traditional knowledge and Indigenous management models to better understand the past and pursue desired futures.

Workshop participants often took issue with questions that involved the terms “traditional” or “management”. For the purposes of this section we use the terms “traditional” or “Indigenous knowledge” not as static types of knowledge, but as Indigenous land-based and intergenerational knowledge [40]. Local knowledge generally refers to knowledge that has been situated in a particular landscape by typically non-Indigenous communities [172]. Iterations of the concepts of traditional knowledge have gone through arduous critiques and many anthropologists use these terms carefully and in reflexive fashions—the commodification and obfuscation of local or Indigenous knowledge is considered in the discussion section, see also [167,168,173,174].

Some participants also took issue with the term “management” because of the association with hierarchical state sanctioned management agencies that have generated negative connotations of the word among local or Indigenous communities. Lertzman’s [175] broadly encompassing treatment of management systems, which includes the sum of actions, goals and objectives, legitimized by social norms, institutions, and actors involved, has been adopted. Participants, particularly Indigenous scholars, challenged the use of the term “resource” (widely used both in social sciences and ecology) as its connotation leads us to think of ecological relationships as dichotomous, hierarchical, and extractive, and called for other ways of describing the world that may help us question such narratives.

### Methods and applications

Methodological practices tend to distinguish historical ecology from other similar research programs and disciplines like environmental history and environmental anthropology. The development of historical ecology in ecology predates the anthropological trend [6]. Ecologists have developed methods for gauging current human impacts on the environment, while archaeologists are able to reveal the timing and extent of specific human activity at the landscape scale and over deep timespans [15,38,176]. The triangulation of multiple kinds of sources and explorations of residual dissonance between different “archives”, actors and ontologies of the natural and human world, are similarly defining characteristics of this field of scholarly endeavor, as the next set of questions highlight.

39. How can modeling of social-ecological scenarios be better developed to incorporate various datasets relating to the past (e.g., paleoenvironmental, historical, archaeological)?

40. How can historical ecology contribute to archaeological investigations of ephemeral sites? (E.g., Sites that reflect activities at a scale that is difficult to detect yet reveals fine-grained temporal records.)

41. How can we see and understand gendered relationships to foodways, past and present? (e.g., food and food systems operating in dynamic socio-cultural environments connected to issues of health and nutrition, livelihood security, labor and power divisions, and cultural and biocultural renewal).

42. What unique contributions might historical ecology make to emergent cross-disciplinary conversations about the Anthropocene?

43. How do we assess and address changes in religious/spiritual interpretations of landscapes?

44. How do land and resource management practices affect nutrient and water cycling in different ecosystems?

45. How can we differentiate between natural and human-mediated range expansions for plants, animals, and other organisms?

46. How can historical ecology engage with Indigenous and local oral traditions that may incorporate diverse spatial and temporal scales?

Questions 41 and 43 address the religious, spiritual, and engendered aspects of landscapes that affect how they are managed [39,177]. These questions could provide new insights to ecologists who may not consider the more subtle or nuanced human activities that shape landscapes [14,178–180]. For decades, anthropologists have wrestled with how to understand and study the relationship between cultures and their biological worlds e.g. [56,181–183]. In the course of this research, many have come to appreciate not only the physical remnants of these interactions, but also the cognitive and emotional experiences [39,184].

Historical-ecological research produces new insights into the relationships between people and landscapes over long time periods, but it requires a careful interlacing of research methodologies from various academic disciplines that incorporate cultural, historical, linguistic, biological, and environmental data [185,186]. Ecological principles and techniques generate strong data that can be used to test hypotheses and build rigorous models to track the subtle changes humans enact on the landscape [187–189]. Question 40 recognizes the ability of historical ecology to integrate proxy markers from multiple disciplines to locate and delimit ephemeral archaeological sites [190], the study of which can further build on understandings of human- environmental transformations.

Although Question 39 highlights the caveat of over-selling and over-representing models, these still provide valuable frameworks for interpreting data and simulating various potential outcomes and are instrumental to many research programs [191,192]. Indeed, van der Leeuw and colleagues [193] have noted that thoughtfully constructed, well-populated models of human eco-dynamics are a necessary component of understanding human-environmental systems at diverse timescales. For example, Question 45 was important for workshop participants who studied ancient transplanting and human-mediated range expansions of important plant species e.g. [194]. Kraft et al. [195] use species-distribution models to support genetic, archaeological, and paleobiolinguistic data to track the domestication of peppers (*Capsicum annuum*). Likewise, Anderies and Hegmon [196] have presented models for understanding human migration and resource use across multiple temporal and spatial scales in the Mimbres region of the American Southwest.

Connecting ecological data (e.g., soil nutrient levels) to anthropogenic actions (e.g., fertilizing and mulching) requires a deep cross-pollination of methodologies (Question 44). In Iceland, Adderley and others [197] have modeled the manuring necessary for Norse settlers to achieve the desired level of home-field productivity when colonizing landscapes. Bean and Sanderson [198] have modeled the effects of historical Indigenous fire regimes on ecosystems in Manhattan, New York.

The methods and application section reflects the broad disciplinary reach of historical ecological research and presages our desire to connect all relevant data to understanding long-term human ecodynamics. As Question 42 suggests, many historical ecologists are interested in contributing to broader conversations about change in contemporary human-natural systems and our arrival in the Anthropocene [32,199]. The compelling integration of data from a multitude of historical and paleoecological research traditions remains one of the most important challenges in archaeology and historical ecology [23], (also see section 4.2 Multi-Scalar, Multi-Disciplinary).

### Communication and policy

Broader outreach requires effective public communication, and it is no secret that academics and scientists are not always the best at engaging audiences outside their chosen fields. It is increasingly apparent that quantitative results do not “speak for themselves” [200], and that practitioners need effective measures to communicate research. Workshop participants felt strongly that historical-ecological research should have an engaged approach, understanding that communication and policy is critical for applied research to be implemented. There is still a widely perceived gap between scientific data and priority information for policy makers [20], as explored in our last group of questions.

47. How can we develop evidence-based frameworks that highlight and overcome the problem of shifting baselines by incorporating long-term archaeological and historical data into contemporary policies and governance?

48. How can we better integrate heritage management laws and policy with those of natural resource management and conservation?

49. How can policy makers, resource managers, and researchers develop respectful, committed, and transparent partnerships with Indigenous and local communities beyond the lifespan of a typical project?

50. How can historical ecology be made relevant for education and built into curricula?

Question 47 highlights the value of generating data of actual utility for policy makers. Two such examples are the re-assessment of ecological baselines for herring fisheries [201], and reviewing reference conditions in the landscapes of the North American Southwest e.g. [46]. Yet there is a caveat to Question 47; historical ecology and other “usable past” approaches can be vulnerable to reductionist narratives of the past [174,202]. This is as true for policy as it is for museums, education, and other curatorial approaches to history [203]. It is acknowledged that the entirety of a particular historical interpretation is not equally accessible or translatable into a policy realm [204]. Recognizing this is crucial to research projects that set out to apply time-series data to advise on contemporary issues [205,206]. Policy should be informed by the amalgamation of diverse data sources and interpretations that are continuously iterated, with the help and consent of all communities involved e.g. [207].

Existing divisions between management of heritage and environmental resources are also particularly problematic. Historical ecology provides solid arguments for fusing heritage and environmental management policy—Question 48 was the highest-ranked question during the final survey exercise. This builds on the recognition that the erasure of humans from a landscape is not necessarily good for ecosystem conservation or associated human communities e.g. [208–210] and that climate change and environmental management (or lack thereof) strongly impact heritage resources [211,212]. Effective policy requires respectful partnerships to develop at the intersection of government, resource managers, researchers, and Indigenous and local people. The need for a commitment to bettering relations between diverse communities in a heterarchical and respectful way was recognized during the priority-setting exercise (Question 49).

Question 50 highlights a facet of communication that transmits historical-ecological knowledge through institutionalized education (e.g., the Global Environmental History MA program at Uppsala University). One potentially effective method for increasing meaningful student interest in the sciences is through project-based curricula or “teaching by phenomena”, which entangles human and environmental elements in a single teachable event or landscape [213].

Teaching historical ecology has the potential to empower others to use knowledge of the past. It also allows for the identification of environmental problems, encourages informed discourse, and supports the development of consensus-driven policy. Participants of the priority-setting exercise all shared a desire for continued engagement with the fluid research program of historical ecology as a way of interpreting the past for the benefit of coming generations.

## Discussion

### Traditional and local knowledge

For millennia, people have been tied to their landscapes through practical experiences and complex sets of environmental and cultural knowledge. As historical ecology navigates multiple iterations of time and space, and seeks to strengthen the breadth of a still-emerging field, traditional and Indigenous knowledge bases are valued as dynamic information sources that can transform or complement Western science traditions. Indeed, the role of ethnographic and ethnohistoric data and engagement with oral historical accounts was a crucial component in most participants’ research tool kits. However, despite the widespread use and celebration of traditional, local, and Indigenous knowledge, many participants felt that our questions should also reflect the global power relations inherent in our work. For example, the legacies and ongoing effects of Western/European colonialism are of particular significance in considering of the complexities of global resource management.

While scholars have long recognized that multi-generational local knowledge systems are a key foundation of successful management e.g. [214–217], it is also important to recognize and discuss the marginalization of Indigenous and local communities from management decisions e.g. [218].Traditional knowledge or, for example, traditional ecological knowledge (TEK), has long been used as anthropological currency in resource development [170] and has been criticized as such [219]. In 1984, a working group on TEK grew out of a symposium hosted by the Commission on Ecology of the International Union for Conservation of Nature and Natural Resources (IUCN), which resulted in several publications and eventual proliferation of the use of the term TEK [220,221]. Since then, TEK has been subject to extended debates in sustainable development and international conservation. The (mis)-appropriation and decontextualization of TEK, particularly in the context of mitigating facilitating industrial development has often had negative impacts, on both the purported resources targeted for management and the communities who subsist and rely on those same resources [167,174,222].

Most participants agreed with the inherent complexity of engaging with Indigenous and local knowledge and yet also agreed that these difficulties in no way negate or invalidate the benefits of doing so. These shared opinions capture insight into the long and complex political landscape to be traversed in order to achieve meaningful collaboration between Western science and local and Indigenous knowledge as a fundamental pillar of historical ecology.

### Eroding boundaries

The results of the question-setting exercise and deliberative process reflected an increased awareness that (1) long-term eco-human dynamics have the potential to be better understood through engaging in multidisciplinary, historically oriented research; (2) there is a surge of interest in applied research; and (3) the boundaries between natural and social sciences are seemingly beginning to erode.

First, our knowledge of local and community-based resource use and landscape management practices is largely derived from ethnographic and historical data. We know relatively little about longer-term (i.e., centennial and millennial) developments of resource use and management systems and the legacies they have created for contemporary ecosystems e.g. [39]. One reason for this lack of clarity is that traditional and locally based practices can mimic natural ecological processes (such as native plant management), and thus the histories of such interactions can often be difficult to detect in the archaeological and paleoecological records [179]. Understanding the mosaic or meshworks of ancient burning, farming and other secondary landscape transformations, e.g. [3], requires strong empirical methodologies. Such research focuses on generating scientific data from multiple disciplines and didactic insights about ethics, politics, conservation, science, destruction, and tradition. By tracking such variation, historical ecologists aim to create a larger picture and broader context for evaluating transformation, adaption, innovation, and social and ecological risks.

Another unifying theme among participants was that historical ecology encourages applied, change-oriented research. The motivations behind early anthropological historical ecology research were born from a context of increasing concern for understanding climate change and land-use governance. A focus emerged on tracking temporal elements of climate change and climate variability [223] while actively recognizing the impacts of human land-use strategies [224].

Historical ecology currently has many configurations, in multiple institutional and disciplinary settings, and not all research and or data collection efforts are directed toward social justice and environmental programs. However, a consensus viewpoint is that an applied historical ecology must act in service of, and consider, research that has wider socio-environmental relevance [9,165].

## Conclusion

The great environmental crises of the twenty-first century will require diverse knowledge sets and the cross-pollination of multiple scientific disciplines to generate innovative solutions. Anthropologists have long struggled with how to conceptualize many types of knowledge (Western, scientific, Indigenous) and have come to recognize the ontologies of “many worlds” (not “one world, many views”) e.g. [225,226], a problem also relevant for historical ecology [227,228]. Landscapes are constituted by individuals and their repetitive actions, where relations with other people and with nonhuman entities, including built landscapes, technology, plants, animals, and others [229–231], interact at varying spatial and temporal scales. In historical ecology, a relational landscape approach recognizes that humans live in animated and continually emergent landscapes, a recognition which opens the field for varying and inclusive perspectives, see also [232].

While the initial goal of our exercise was to identify priority research questions relating to the emergent field of historical ecology, workshop participants decided to be less insular, realizing that the developments of historical ecology and its associated expressions vary across disciplines and geographic locales of practice. However, our anthropological and archaeological focus of self-selected respondents is indicative of one aspect of the surging influence that historical-ecological research is developing across multiple academic disciplines. Taken together, these series of highlighted research questions strengthens the basis for collaborative and mindful research to better understand the interrelated entanglements of people and environment over the course of human history.

## Supporting information

S1 Table List of participants from Uppsala and Vancouver workshops. (DOCX)

S1 File Complete list of all questions submitted to New International Community for Historical Ecology (NICHE) organizers during the crowdsouring portion of research. (DOCX)
