# Supplementary material for: Anthropological contributions to historical ecology: 50 questions, infinite prospects
Source: PLoS One. 2017 Feb 24;12(2):e0171883. doi: 10.1371/journal.pone.0171883 (PMC5325225; doi:10.1371/journal.pone.0171883)
Supplement: S1 Table — (DOCX) [file pone.0171883.s001.docx]

**S1 Table. List of participants from Uppsala and Vancouver workshops**

Uppsala Workshop

Kay, Andrea

Shoemaker, Anna

Ekblom, Anneli

Pas Schrijver, Annemiek

Crumley, Carole

Armstrong, Chelsey Geralda

de Bont, Chris

Quintana Morales, Eréndira

Hoffman, Erika

Weiberg, Erika

Mtetwa, Ezekia

Humphrey, Glynis

Kjellberg, Joakim

von Hackwitz, Kim

Smiarowski, Konrad

Papaioannou, Kostadis

Berglund, Kristina

Duncan, Lindsey

Hicks, Megan

de Haas, Michiel

Ramsey, Morag

Petek, Nik

Boles, Oliver

Lane, Paul

Sinclair, Paul

Kariuki, Rebecca

Hughes, Ryan

McGovern, Thomas

Vancouver Workshop

Anderson, E.N.

Angelbeck, Bill

Astudillo, Fernando J

Awasis, Sakihitowin

Boles, Oliver

Burley, Dave

Buckley, Sarah

Chastain, Stephen

Crumley, Carole

Eagan, David

Giesz-Ramsay, Tracy

Gibbons, Kevin

Ibragimow, Aleksandra

Poolaivray, Gregor

Johnson, Leslie Main

LeCompte, Joyce

Lee, Lynn

Lepofsky, Dana

Marks-Block, Tony

McCrea, Grace

McAlvay, Alex

McKechnie, Iain

Mtetwa, Ezekia

Nabess, Carly

Novick, Adam

Rodrigues, Antonia

Johnston, Buffy

Petek, Nik

Quintana Morales, Eréndira

Purcell, Emily

Royle, Thomas C.A.

Sabbath, Sunday

Savo, Valentina

Shoemaker, Anna

Royle, Tom

Stahl, Peter

Tobiasz, Mary Lynn

Valadares, Desiree

Vamosi, Jana

John Vandergugten

Walshaw, Sarah

Wolverton, Steve

Zimmermann, Mario
