# Supplementary material for: Anthropological contributions to historical ecology: 50 questions, infinite prospects
Source: PLoS One. 2017 Feb 24;12(2):e0171883. doi: 10.1371/journal.pone.0171883 (PMC5325225; doi:10.1371/journal.pone.0171883)
Supplement: S1 File — (DOCX) [file pone.0171883.s002.docx]

**S1 File. Complete list of all questions submitted to New International Community for Historical Ecology (NICHE) organizers during the crowdsouring portion of research.**

1. What can we learn from local "traditional" knowledge in development for biodiversity

of landscapes?

2. How are historical legacies of past land-use reflected in current ecosystems (species

distribution, ecosystem functioning)?

3. How do we apply historical knowledge of resource/natural management into fair and

just policy?

4. How did societies past adapt to sudden change and how can we learn from it?

5. How can historical ecology and knowledge of past and present human-nature

relationship receive the respect it deserves in sustainable development policy, combining

it with ethical economic-political perspectives in development?

6. How the history of landscape or forest history can help us to understand and mitigating

climate change?

7. How can the different human process shaped through time and cause to climate

change?

8. How can we identify landscape patterns and biodiversity by studying historical

ecology

9. Can we develop a new environmental model by looking the past history of ecology?

10. Can we develop new adaptability method and farming model by looking back in to

the history of ecology?

11. When did the Anthropocene begin?

12. How much was the landscape changed by the onset of agriculture?

13. Is there a correlation between ecological and social changes in the past?

14. How resilient were human systems and ecological systems in the past?

15. Why do we have this anthropocentric western culture bias, according to Lynn White,

that lead to today ecological crisis?

16. What were plagues (Justinian & Black death) tremendous impacts?

17. What is the central role of energy in anthropology?

18. Why do anthropologists and economic historians disregard energy in global history,

when the central role of energy in today crisis is so obvious?

19. What were in the last millennium previous "limits to growth", and resources

constraints?

20. how do the short-term, medium-term and long-term aspects of human-land

relationships interact?

21. When did humans start to have an impact on land cover and biosphere at the level of

biomes?

22. How do extinction debt and migration credit influence current ecologies?

23. When did the first cultural landscapes develop in Europe?

24. How predictable are the effects of human changes of environment?

25. What could be the role of power and ideology in a better understanding of human

ecosystems?

26. How do we conceptualize nature and culture in such a way that we avoid the

traditional dualisms in nature/culture thinking?

27. What is the value of the human niche construction concept for the further

development of historical ecology?

28. How important is the "Anthropocene Debate" for historical ecology?

29. What can we actually learn from past landscape systems?

30. How to deal with different temporal and spatial scales, how to define them & how to

study there interaction?

31. How can historical ecology be more effective for spatial planning and design issues?

32. How can we give the historical ecology a better place in academic training? (as it is a

profound interdisciplinary challenge)

33. How to bring researchers from Europe, America, Asia, Africa together around shared

topics and issues? How to organize debate? A separate "World Conference of Historical

Ecologists"?

34. What are the physical and genetic markers for intentional livestock improvement?

35. How can historical ecologists communicate the potential to utilize our data for

present-day public policy regarding climate change?

36. Can we decide on a widely-accepted definition for 'modernity' and does modernity

correlate to the Anthropocene in any meaningful way?

37. Does historical ecology have to deal exclusively with the longue durée?

38. What can biorefugia or remnant communities/populations tell us about island

biogeography (and vice-versa)

39. What are the limitations of adaptation within a system?

40. What is the influence of environmental resource management and restrictions on the

livelihood of traditional communities and local populations

41. How can we address the importance of culture on conservation biology and on

conservation policies?

42. What has been the role of grazing by large herbivores in the maintenance of various

landscapes?

43. How do we evaluate environmental resilience when we are considering contemporary

alteration, impact, or influence of human activities on landscapes?

44. Can Historical Ecology be a bridge between the Intangible Cultural Heritage and the

Landscape Ecology knowledge?

45. How can Historical Ecology help us to comprehend the natural dynamics and

processes of adaptation and resilience induced by human activities?

46. How can Historical Ecology help in the deconstruction of the so excluding 'untouched

nature' ideal that still drive forces many conservation efforts worldwide?

47. What is the potential for phylogenies to be used as a tool for predicting the edibility

of plants, and finding wild crop relatives for plant conservation?

48. What is the relationship between the diversity in diet of food plants and the nutritional

adequacy of individuals and communities?

49. What is the rate of evolution of secondary metabolities of plants in order to improve

estimates of their medical potential as well as explore the mode of action of companion

planting?

50. What is the degree to which different communities have found uses for local plant

biodiversity?

51. How can DNA barcodes be employed to improve estimates of local and regional

biodiversity with phylogenetics?

52. How does biological diversity turnover between areas (betadiversity) relate to cultural

diversity?

53. How do we use technology to visualize and communicate time?

54. How can historical ecology be made relevant for education?

55. Can we use species range extensions to infer human transplanting of plant species?

56. How does biological diversity turnover between areas (betadiversity) relate to

sustainability in culturally relevant species?

57. What is and was the impact of settler colonialism on Indigenous environmental

systems?

58. How settler colonialism has impacted social and natural environmental systems

including indigenous management of resources?

59. How communities value and manage the natural resources that surround them?

60. If traditional management principles and practices design and are influenced by the

natural environment around, building unique landscapes (in a broad sense) what about to

design human/nature relationship "conservation" areas?

61. The invisible parts of ecosystems: how and what can we find out about events that

happen to rarely to be considered regular parts of ecosystems?

62. How will the field of epigenetics influence our understanding of early crop

domestication?

63. How to merge the traditional knowledge, principles and values of communities, and

the economic development, well fare and prosperity?

64. How to include communities way of life and demands in local and regional economic

development strategies in developing countries?

65. How can a biologist interested in well fare and conservation biology still do without

social sciences fellows?

66. How did the colonization of British Columbia affect populations of mountain goats

and wool dogs thereby changing Coast Salish wool-woorking practises?

67. How do people working in fibre adapt to changes in what fibres are available and

methods for using fibres?

68. How has anthropogenic fire influenced ecosystem functionality and fire regimes?

69. How has anthropogenic fire affected landscape ecology?

70. How has fire suppression/exclusion altered the decision-making related to cultural

burning in contemporary indigenous communities?

71. How does contemporary anthropogenic fire affect indicators of socioeconomic and

cultural vitality in indigenous communities?

72. How do different histories of dispossession and land tenure affect anthropogenic fire?

73. What is the role of the built environment in the social interaction making up how

cities function?

74. How is resource and environment management part of everyday urban life?

75. What can we learn from archaeological examples of ancient urban landscapes

representing a great variety of urban traditions about how we can improve urban life and

design going into the future?

76. How do the variety of (large) open spaces within ancient tropical urban landscapes

function in everyday social life and urban development?

77. In which ways can we make archaeological and other historical humanities findings

relevant to socio-environmental processes taking place today?

78. How can historical ecology create an intervention in the human-environment

paradigm that ensures the critical, contextual and reflective use of the full breadth of data

on past human behaviour within their social and natural environment?

79.How can historical ecology be put to use in improving planning and (urban) design for

both socially and naturally sustainable modes of living/inhabiting, and how can it inform

processes of development towards improvement thereof?

80. How can historical ecology be applied as a research program in area like medical

anthropology?

81. Does historical ecology always have to include humans?

82. How can we use the Amazonian Dark Earth’s (terra preta) to better understand

anthropogenic soil accumulations in other parts of the world?

83. How can landscape change be understood as a key feature in human health?

84. How can the historical study ethnoepidemiology produce relevant data to a medical

ecological perspective?

85. Will soil micromorphology become more important for understanding human-scale

landscape presence then traditional archaeological strata?

86. How can the sickness-health relationship be historically understood in plantations

landscapes in the Caribbean area?

87. How do ethnosemantics relate to sickness from a historical perspective?

88. What is sustainable development?

89. What behaviour can be traced to explain resistance to adapt to climate change?

90. How can a paleoecological perspective sharpen the focus of biodiversity management

and conservation policy?

91. How can paleoecology and historical ecology data / insights be made explicit, and

their data sets made accessible and amenable to management-centred purposes?

92. Can a policy–ecology–environment dynamic framework be developed to integrate

long-term variability, thresholds and limits of acceptable change in ecosystem service

provision?

93. Can ecosystem flickering responses can be found in palaeoecological records that can

be used to distinguish flickering signals from normal background variability?

94. How can we use ancient and modern genetic data to infer past management practices?

95. To what degree, does the genetic structure of modern plant and animal populations

reflect past management practices?

96. How reliable is the archaeological and palaeoecological data we use to address

historical ecology questions (e.g., How accurate are the taxonomic identifications

archaeologists assign to faunal and floral remains?)?

97. What factors influence the reliability of this data? How can we test the reliability of

this data?

98. How can we determine if now-exotic floral and faunal species identified in

archaeological contexts reflect long-distance transport or changes in a species'

distribution?

99. Under what conditions does resource depression occur?

100. When did humans begin to exploit and manage aquatic landscapes?

101. How can we overcome institutional structures (e.g., University departments centered

around disciplines rather than research interests, degree specialization, predominance of

single discipline-centric journals and conferences) that inhibit the cross-disciplinary

research and collaboration that characterizes historical ecology?

102. When did human activities begin to have a measurable effect on local and global

climatic systems?

103. How can we best estimate the past abundance and variability of species from their

abundance in zooarchaeological assemblages?

104. To what extent do human behaviours (e.g., preference for certain species, harvesting

technologies and practices) alter these estimates?

105. Why have some cultural systems collapsed when faced with environmental change

while others have prospered?

106. What factors have made some cultural systems more resilient to environmental

changes than others?

107. How have humans mitigated temporal and spatial variation in the abundance of

resources?

108. How can we differentiate between natural and human-mediated range expansions?

109. To what extent have past human activities driven changes in the morphology and/or

behaviour of plant and animal species?

110. How has European colonization structured the current vegetation patterns?

111. How did natural disturbances and Indigenous land management practices structure

pre-colonial vegetation patterns?

112. In the context of global change, are historical references relevant for ecological

restoration?

113. How to develop management models consistent with the functioning of ecosystems?

114. How is nature conservation framed, motivated and implemented?

115. What actors are involved in establishing protected areas and do they consider

landscape and conservation history and future?

116. How do actors and actor-groups involved in establishing conservation areas

motivate and envision the establishment of large-scale trans-frontier conservation areas?

117. How to balance the interests of people with needs to protect biodiversity and

wildlife for current and future generations?

118. How does the socio-political history of nature conservation affect current attempts to

establish protected areas?

119. How have conservation projects in the past been carried out and what factors

contribute to the failure/success of these practices? How can lessons from this be

incorporated to and accounted for in future conservation practices and policy?

120. In what ways can nature conservation be part of increasing resilience and climate

change adaption?

121. How are conservation landscapes defined, described and framed, in past, present and

future and how can diverging actors and their interests in nature conservation be

negotiated for mutual sustainable futures?

122. How can past and current local ecological practices and knowledge be incorporated

and translated into policymaking?

123. How can stakeholders in natural resource management and conservation projects be

involved in an equal way?

124. And how can past practices in natural resource management be reconciled with

current initiatives and projects?

125. How can historical ecology contribute to solve current and future issues of global

food security (sovereignty?) - both in terms of social organization and sustainable

ecological practices of food security?

126. How can we best communicate historical ecological knowledge and connect it to

current day practices - how can we exemplify and promote the importance of analyses of

the past to the benefit of more sustainable futures?

127. What are the best statistical methods to integrate data on human distribution, climate

reconstructions, land use patterns in historical time, and political instabilities.

128. Finding regularities (scaling laws) in statistical patterns of early human dynamics

that can be extrapolated to areas with no records.

129. Is it possible to reconstruct early-human past movements using range distribution

inferred for large mammals using species distribution models?

130. What data standards should we develop to consolidate relevant information en a

consolidated (open source) database?

140. What are the critical factors that allow human populations become more decoupled

from environmental constraint?

141. How does a climatic perturbation influence human behavior?

142. What are the signatures of past climate stability?

143. How to relate both past and present human-environmental narratives to inform

applied management approaches?

144. How to quantify human actions and/or roles/ or the social dimension in ecological

models?

145. How are subjectivity and differential human values adequately dealt with in models?

146. How do I encompass the multiplicity of different viewpoints, including indigenous

knowledge into the management of natural resources?

147. How do I bridge the gap between subjective and objective knowledge to address

present-day ecological uncertainties?

148. How to encourage the incorporation of Indigenous knowledge into Western science

as an approach for addressing present-day environmental issues?

149. How do we quantify social data to enhance applied management approaches in

unpredictable and uncertain social-ecological circumstances?

150. How do we constitute humans as integral parts of ecosystems rather than as negative

disturbances?

151. How do we conceptualize humans as one of many species in an ecosystem rather

than protagonists?

152, How do we encourage a continuum of research projects focused on indigenous

community knowledge beyond a typical projects lifespan?

153. How do we standardize the outputs of social data to complement ecological data in

models?

154. How do we encourage western scientists to acknowledge data/knowledge collected

in indigenous communities as binary authorship?

155. How could we use Historical Ecology to challenge current ecological theoretical

paradigms which limit addressing solutions’ in the Anthropocene period?

156. How could we use the Historical Ecology platform to raise awareness about the

importance of writing research critically that openly and constructively addresses the

embedded western positivist approach to science?

157. How to link between past and present-day human activities to address current

environmental issues?

158. How do we relate both past and present human-environmental narratives to inform

applied management approaches?

160. How do we encourage re-creating, re-organizing and re-transforming research

narratives perceived from a multiplicity of viewpoints into an agenda of open debate in

Historical Ecology?

161. How have the socio-ecological systems of temperate rocky reefs in BC changed

from prehistoric times to the present (focus on people, sea otters, urchins, abalone and

kelp)?

162. How can we best integrate multiple sources of knowledge to generate lines of

evidence that help us build understanding of system changes over long time spans?

163. How can we best engage with First Nations and local communities to respectfully

incorporate traditional knowledge and local knowledge into historical ecology projects

that are specific to place?

164. How can historical ecologists best work with other disciplines (e.g., archaeology,

paleogeography, historians) in effective multidisciplinary teams to address place-based

projects, and use specific results to build a broader understanding of changes over larger

geographic areas and long time spans?

165. How and why have species abundances and demographics changed through time

and how has human dependence adapted to that change?

166. What can we learn from First Nations traditional management systems in the past to

inform innovative and effective future management?

167. How should academics engage respectfully and meaningfully with First

Nations/aboriginal communities to conduct place-based historical ecological research?

168. How do we gauge/measure changes in cultural/religious interpretations of

landscapes

169. How does the advancing colonization by the global market change social relations

and cultural/religious interpretations of landscapes in communities that could be seen as

on the outskirts of "development".

170. How does gradual or rapid change from a production system oriented mostly

towards subsistence towards a market orientated system show on the landscape?

171. How the meaning of a changing environment is affecting people's ideas and

behaviour towards nature and other humans

172. How modernity is shattered and re-articulated through environmental problems

173. How have societies "environed" nature in different ways over time?

174. How does the meaning of nature change and what factors influence this meaning

most?

175. What is the relationship between economic systems and the ecological system?

Especially the relation between economic policy (growth, monetary policy, interest) and

environmental degradation.

176. How does distance affect our sense of responsibility? Can a localized economy be a

way to decrease our negative impact on the environment?

178. What are the main challenges and possibilities for local economies?

179. Which lifestyle (rural vs. urban) is the most sustainable? How do the calculation

change when consumption is included in the footprint of the consumer and not the

producer?

180. How do we our translate research into policy? Especially integrating research results

from historical ecology into the field of economics.

181. What is the impact of the metal industry on the environment?

182. What were the strategies of human natural resource exploitation and did

management strategies exist?

183. How did ancient people choose raw materials; according to their qualities and/or

their availability or both?

184. Did ancient people change their habits of exploitation in the course of operation of

their iron smelting workshops?

185. Is it possible to relate vegetation changes to the overexploitation of woody species

for ironworking or do they simply reflect oscillations of climate and land use intensity?

186. What natural resources are used from the sea and land areas in the economy of the

ports?

187. How were ports sited in relation to siting the physical and biological environment of

the coast?

188. How can we document arborealculture archaeologically?

189. What species were exploited surrounding ports and how did this effect the

environment?

190. How have societies in the past adapted to environmental change such as harbour

silting and changing seasons, and unpredictability from tsunamis and cyclones?

191. Do we need more detailed lower-level understanding before expanding to bigger

scales or issues?

192. What can ancient texts tell us about attitudes toward the environment?

193. Anthropogenic enhancement of the environment, either intentional or unintentional

– is the emphasis on this relationship always destructive?

194. How can archaeologists make their results relevant to modern environmental and

climate issues?

195. To what extent is Historical Ecology defined by methods?

196. How do we encourage more interdisciplinarity, to increase the methods, data and

perspectives available to us?

197. How do we communicate effectively with those outside of

archaeology/anthropology (including policy makers)?

198. What happens to anthropogenic landscapes post-occupation? How can these

perspectives be applied to modern land use or planning?

199. How have ancient activities impacted the ecological trajectory at a site?

200. Do we yet know enough information about relationships at the smaller scale to be

working at larger scales?

201. Is prehistoric land use a viable factor for determining future management decisions?

202. What is the best way for a researcher to identify themselves as working with

historical ecology (in the absence of defined criteria), in order to establish an effective

network of researchers working towards common goals?

203. What is the role of prehistoric landscape modification to identify the baseline/steady

state that should inform environmental management decisions?

204. How to recognize patterns of prehistoric land use by hunter-gatherer people to more

effectively locate sites for excavation or preservation?

205. How to use archaeology and anthropology to compile traditional ecological

knowledge, and then test this body of knowledge for its applicability in contemporary

issues?

206. What can genetics and ecology of domestication teach us about land use and marine

resource use, especially in the context of climate change induced scarcity?

207. What role did humans play in the extinction of Pleistocene megafauna? Are the

dynamics of these extinctions different from the ones we see today?

208. Is it possible to create enforceable treaties and laws dealing with forces that work on

timescales beyond those of politics and markets?

209. What is the proper way to incorporate the "techno-fix" idea into models?

210. How do novel ecosystems come into being?

211. Should abandoned places such as Pripyat, Ukraine or Centralia, USA be treated as

living experiments in natural succession of abandoned areas?

212. What are the proper ways to use traditional ecological knowledge in a modern

decision-making framework? Should there be a concerted effort to include this in

scientific literature?

213. Are dissimilar environmental impacts indicative of cultural values?

214. How do we (can we) quantify non-material cultural aspects in the material

signatures of the past?

215. What are the environmental and archaeological signatures of epidemics?

216. What mechanisms are available to merge local variability within a global context?

217. Are cultural values accessible to an historical ecology?

218. How can Indigenous Oral Traditions merge with Historical Ecology? Is it strictly

local?

219. Is there a correlation between social organization and environmental impact?

220. Should historical ecology become its own academic discipline and why?

221. Do we need to include modelling into our studies in order to get a more inclusive

and better understanding of the past?

222. What is the best way to tackle the issue of time and space, to join them into a single

concept?

223. Would a single concept of time and landscape/space (time-space singularity) allow

for a better understanding among disciplines of these concepts, thus allowing different

disciplines to better cooperate?

224. Is historical ecology in need of a text book-type paper, which explains the different

time scales and how they are used in disciplines, and which will teach new students how

to best use different time scales for their own research?

225. How can we best incorporate historical ecology in future urban and landscape

planning for a better preservation and greater biodiversity?

226. Most historical ecologists are working in archaeology or are tightly related to it.

Should archaeology continue to be the "central" discipline or the node to/through which

other disciplines connect? Or should we try and build historical ecological narratives with

each discipline acting as a node/connecting point during one point in time during the

research?

227. Should historical ecology train people to be interdisciplinary in their studies, or

people who are mono-disciplinary in practice but have an good and applicable

understanding of other disciplines?

228. What is the best definition of 'landscape' so that it is applicable across disciplines?

229. Which geographical regions are currently lacking historical ecological analyses?

230. How can historical ecology encourage a sustainable farming practice which is rich in

species being cultivated, and which also does not endanger the biodiversity of a region?

231. Although historical ecology studies past landscapes and environments for a more

sustainable future, most studies do not look into the future. Is there a possibility for

historical ecology to expand its temporal scope into the future through modelling and the

application of scientific methods?

232. Do we always take into account what we are conserving, the politics behind the

conservation, for whom we are conserving, and what the implications and consequences

of conservation projects are?

233. What have we learned in the past decades from historical ecology? What has it been

doing right/wrong?

234. How can we best disseminate knowledge to the people in the area we are working

with? How can they employ that knowledge?

235. Are there significant diachronic correlations between the health of humans and the

vitality/integrity of the ecosystems they rely upon?

236. How do you integrate data from various disciplines to represent a long term record

of biodiversity and natural resource use?

237. What are the key cultural transitions that transform the way societies engage with

their surrounding landscape?

238. To what extent can current models of natural resource use help us understand the

past?

239. How does social identity affect the way individuals and groups exploit natural

resources and integrate them into daily life?

240. How do environmental and cultural phenomena influence the role of natural

resources in societies?

241. What is the role of historical ecology researchers in the conservation of cultural and

natural diversity?

242. How can researchers effectively communicate their research to the general public

and with what purpose?

243. How do expectations of climate change affect responses to climate change and how

does predictability and knowledge of periods of climate change mitigate the risks?

244. How can local knowledge be justly and responsibly incorporated into research and

policy to address present-day environmental solutions?

245. How do assumptions about the local or universal nature of human-environmental

interactions influence research in historical ecology?

246. How can historical ecology contribute to issues of global food security

(sovereignty?); for example, shifting the focus away from global food shortages and onto

inadequate global food distribution?

247. How could we use historical ecology to challenge current ecological theoretical

paradigms which limit addressing solutions in the Anthropocene period?

248. How do people globally engage with historical ecology? How is it practiced as a

research in geographically diverse locales, by researchers from a multitude of disciplines?

249. What are the ways that we discuss and analyze landscapes as memories, as locations

of human action, as political boundaries, or as ecosystems?

250. What are different heritage traditions as expressed by archaeologists, indigenous

peoples, and governments?

251. What is the history of heritage site conservation policy, both in British Columbia

and Western settler societies?

252. Does heritage site conservation practice conform to policy?

253. What makes for effective heritage policy? How does heritage policy relate to

broader issues?

254. How can we define HE in terms of other disciploines, history, ecology geography

and systems analysis

255. How best can we conduct HE research in a multiscalar multi temporal frames of

reference

256. Is it necessary to distinguish between archaeological and ecological data?

257. Is pastoralism inherently environmentally-destructive?

258. How is pastoralism understood by land-use policy makers?

259. Can there be an historical ecology of the prehistoric period?

260. How have subsistence decisions affected resilience to past environmental change?

261. Can we look to the ecological data as a surrogate for the archaeological record,

where the latter is missing or ill-defined?

262. Do current perceptions of pastoralism and its impacts by policy makers reflect longterm

past realities?

263. To what extent do subsistence decisions influence resilience to environmental

change?

264. How do community perceptions of environmental change differ globally and to what

extent can such differences be understood historically?

265. To what extent do environmental stress events influence long-term subsistence

choices?

266. What are the ecological impacts of anthropogenic soil formation?

267. Can ecologies observed at the local scale be transposed to regional and global

scales? How?

268. What is the relationship between anthropic environmental degradation and social

complexity?

269. What impact do restrictions on/reductions in mobility have on environmental

degradation?

270. How does the removal or introduction of domesticates (plant and animal) affect

wider landscape ecologies? Over what timescales?

271. By what mechanisms is social identity linked to subsistence? How are these

manipulated?

272. Is the diversification of subsistence strategies inevitable?

273. Does the protection of biodiversity conflict with global food requirements?

274. How can an understanding of ecological history contribute to global food provision?

275. Can there be a historical ecology of the prehistoric period?

276. What is the changing role between natural and anthropogenic landscapes since the

Pleistocene?

277. Are humans inherently destructive to their environments?

278. Is there really such a concept as a "natural ecology" since the Pleistocene?

279. What can historical ecology tell us about the future of the environment given the

ever-growing impact of the Anthropocene in shaping landscape?

280. What role has climate played in shaping biotic communities of the past?

281. How can we make historical ecology truly inter-disciplinary -- i.e, so we really cross

and integrate disciplines/communities/worldviews

282. Why does historical ecology continue to be a relatively marginalized field of

inquiry?

283. How does changes in food sources relate to changes in social structures?

How can we make scientific experiments more culturally relevant?

284. How do food sources relate to social systems?

285. How can we make scientific experiments more culturally relevant?

286. How does experiential research relate to scientific experimentation

287. Can experiential research and scientific experimentation meet each other and

transform the way we learn about and understand the world, the land and the

communities that we are part of?

288. What does it mean to practice sustainability?

289. Is sustainability only for environment? Or can the concept of sustainability, or the

question of what it means to sustain, be applied more broadly to the study of

anthropology, ecology or mathematics?

290. How do epistemologies of sustainability vary across fields?

291. What becomes normatively understood as deserving sustaining?

292. How has the extraction and intensive exploitation of natural resources affected the

culture and ecology of the indigenous peoples?

293. How do the indigenous peoples who live in watersheds perceive the modifications

over their rivers, lands and forests made by non-indigenous people?

294. How do the United Nations’ environmental policies affect the human rights of

hunter-gatherer communities living in the rainforests

295. How is it possible to protect the economies of indigenous hunter-gatherer peoples

from the merchandising of their territories in the global carbon market?

296. What strategies did people use to avoid overfishing?

297. What strategies did people use to mitigate temporal-spatial fluctuations in aquatic

resources?

298. What are the long term strategies responsible for adaptation in a social ecological

system?

299. How do you study change on an island community where spatial controls are

unavailable AND temporal scales are poorly understood

300. How long does it take to quantify human impact on the landscape (e.g., Galapagos

were only recently colonized in early twentieth century – can we understand human

impact on the environment/landscape at this scale yet?)

301. When people move to isolated places – do they adopt a different lifestyle or do they

shape the landscape themselves?

302. Why do different cultural groups in the same bioregions utilize plants in dissimilar

way?

303. What is the role of tourism/temporary visitors on a landscape? How does this effect

our interpretations of a landscape (e.g., Galapagos)

304. What does a pristine forest look like? Does it exist?

305. How do the general public define prisitine? Why do they focus on large fauna versus

invasive species (e.g., large fauna easier to identify, more appealing) – is that an accurate

way to portray “nature”?

306. How do we translate the “landscape scale” to marine ecosystems? Is it even

possible?
